# Effects of Vegetation, Corridor Width and Regional Land Use on Early Successional Birds on Powerline Corridors

**DOI:** 10.1371/journal.pone.0031520

**Published:** 2012-02-20

**Authors:** Robert A. Askins, Corrine M. Folsom-O'Keefe, Margaret C. Hardy

**Affiliations:** Department of Biology, Connecticut College, New London, Connecticut, United States of America; Australian Wildlife Conservancy, Australia

## Abstract

Powerline rights-of-way (ROWs) often provide habitat for early successional bird species that have suffered long-term population declines in eastern North America. To determine how the abundance of shrubland birds varies with habitat within ROW corridors and with land use patterns surrounding corridors, we ran Poisson regression models on data from 93 plots on ROWs and compared regression coefficients. We also determined nest success rates on a 1-km stretch of ROW. Seven species of shrubland birds were common in powerline corridors. However, the nest success rates for prairie warbler (*Dendroica discolor*) and field sparrow (*Spizella pusilla*) were <21%, which is too low to compensate for estimated annual mortality. Some shrubland bird species were more abundant on narrower ROWs or at sites with lower vegetation or particular types of vegetation, indicating that vegetation management could be refined to favor species of high conservation priority. Also, several species were more abundant in ROWs traversing unfragmented forest than those near residential areas or farmland, indicating that corridors in heavily forested regions may provide better habitat for these species. In the area where we monitored nests, brood parasitism by brown-headed cowbirds (*Molothrus ater*) occurred more frequently close to a residential area. Although ROWs support dense populations of shrubland birds, those in more heavily developed landscapes may constitute sink habitat. ROWs in extensive forests may contribute more to sustaining populations of early successional birds, and thus may be the best targets for habitat management.

## Introduction

Open corridors along powerlines have become a prominent feature of landscapes throughout the world, leading to concern about their environmental effects. Most ecological studies of powerlines have focused on potentially negative effects. Collisions with powerlines and electrocution can cause high mortality in some species of birds [1]–[2], and the open corridors along powerlines can fragment forests and other natural habitats, leading to a loss of biological diversity [3], [4]. Relatively few studies have investigated the positive effects of powerlines, and most of these have emphasized the potential role of powerlines as corridors connecting natural areas and consequently reducing the effects of isolation for populations in habitat fragments [4]–[6]. Powerlines can also play a more direct positive role, however, by providing extensive, continuous habitat for species that require low vegetation. The open rights-of-way along utility lines provide habitat for declining species of birds in North America [7], mammals in Australia [8], reptiles and amphibians in North America [9] and insects in North America and Europe [10], [11].

Habitat management on powerline corridors has been emphasized in conservation efforts for early successional birds in eastern North America, where many species that require shrub/scrub habitats have declined in recent decades [12]–[13]. In the northeastern United States (New England south to Virginia and West Virginia), 14 of 27 species of shrubland birds declined significantly between 1966 and 2007 [14]. The primary cause of these declines appears to be the loss of early successional habitat due to regrowth of forest on abandoned farmland and suppression of natural disturbances such as fire, beaver activity and seasonal flooding [15], [16]. Conservation agencies and organizations can create or maintain shrubland habitat, but this requires either expensive, continuous mechanical brush removal, herbicide spraying and/or prescribed burning to prevent the growth of trees [7], or the reintroduction of natural disturbances, an approach that is usually only practical in large natural areas. Consequently, the total area of conservation land maintained as early successional, woody vegetation is relatively small and the future of shrubland birds in most of eastern North America will probably depend on early successional habitat created as a result of economic activities such as timber harvesting and maintenance of open corridors for high-tension powerlines [7].

**Figure 1:**
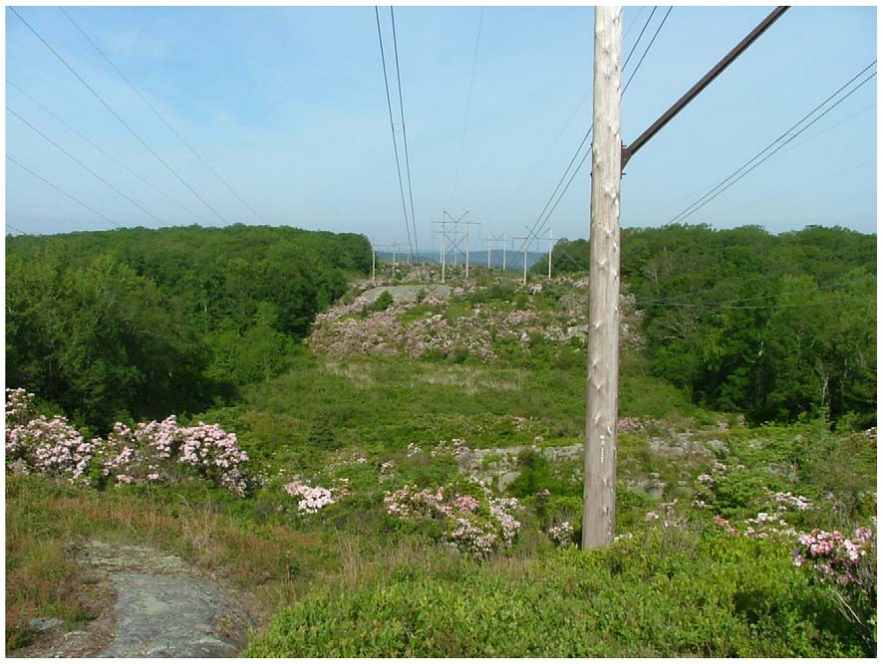
Powerline corridor managed by selective removal of trees to maintain low vegetation in Montville, Connecticut, U.S.

Powerline rights-of-way (ROWs) provide a stable source of appropriate habitat for shrubland birds because large areas of early successional habitat must be continually maintained to ensure that overhead lines are kept free of tall-growing vegetation [17]. Habitat is especially favorable for early successional species where herbicides are applied selectively to target tall-growing trees and invasive shrub species in the powerline corridor [18], (Figure 1). Graminoids, forbs, and native shrubs (especially clonal species) are then released from competition with trees and spread to create a low-stature plant community that is relatively resistant to tree invasion [19]. This management practice was developed under the guidance of plant ecologists in the 1950s as an alternative to repeated broadcast spraying of herbicides along powerline corridors. It is now a standard method of powerline maintenance in much of northeastern North America and has been used at some sites in Australia, but is still not widely used in other parts of the world, where powerline corridors are maintained by frequent mowing or broadcast herbicide spraying [20].

Powerline corridors that are maintained by selective spraying of herbicide to remove trees have a greater diversity and density of birds than corridors maintained by mowing or non-selective, broadcast spraying of herbicides [7], [21]–[22]. This is partially due to the greater abundance of shrubland specialists in the dense shrub cover that results from the selective removal of trees [7]. Several studies have shown that the abundance and nest success of these shrubland specialists are related to plant species composition and vegetation height within the corridor as well as to the width of the corridor [7], [23]. The effect of land use patterns in the region around corridors has only been tested recently, however [23]. Land use patterns may affect the abundance of nest predators and brood parasites that reduce reproductive success of songbirds. Shrubland specialists may be especially vulnerable because they have open-cup nests. Studies of forest birds show that nest predators and brood parasites often have a much larger impact on nest success in agricultural and residential landscapes than in heavily forested landscapes [24]–[25], and some shrubland species may be affected by human development in a similar way [26]–[27].

Our goals were to determine (1) whether an extensive powerline ROW system in southeastern Connecticut maintained by selective removal of trees supports populations of declining species of shrubland birds, (2) whether shrubland birds nesting on powerline corridors produce enough young to sustain their populations and (3) whether the abundance of particular species of shrubland birds is more strongly related to vegetation structure and composition, the width of the corridor, or land use patterns in the surrounding landscape. Our results should indicate how to manage powerline corridors more effectively to provide habitat for bird species that have a high priority for conservation. The results may also be relevant to management of early successional habitats in conservation areas using methods developed and tested along powerlines. Although our study focuses on declining shrubland bird species in North America, the results may be relevant to conservation in temperate woodlands in Western Europe and East Asia, where similar declines in early successional birds have been documented [28]–[30].

## Results

### Bird species detected in ROWs

We recorded 65 species of birds during point counts in ROWs. The 28 most abundant species (those detected at >10 survey plots), including seven species associated with shrubland habitat, are listed in Table 1. The following shrubland species were detected at more than half of the survey plots: eastern towhee, prairie warbler, field sparrow and blue-winged warbler (see Table 1 for scientific names). Also, we observed yellow-breasted chat (*Icteria virens*), a Connecticut-listed endangered species, at two plots. Brown thrasher (*Toxostoma rufum*), a Connecticut-listed species of special concern, was recorded at one survey plot, and two pairs produced fledglings at our nest study site in 2007.

**Table 1:** Frequency (proportion of plots occupied) and average number per plot with standard deviation for bird species detected in >10 survey plots (n = 93) on powerline corridors.

Species	Frequency	Average(St. Dev.)	Associated with shrubland^a^
Eastern Towhee (*Pipilo erythrophthalmus*)^b^	0.81	1.20 (0.92)	Yes
Gray Catbird (*Dumetella carolinensis*)	0.77	1.15 (0.81)	
Brown-headed Cowbird (*Molothrus ater*)^b^	0.54	1.10 (1.52)	
Prairie Warbler (*Dendroica discolor*)^b^	0.73	1.06 (0.88)	Yes
Field Sparrow (*Spizella pusilla*)^b^	0.59	0.85 (0.87)	Yes
Common Yellowthroat (*Geothlypis trichas*)^b^	0.66	0.82 (0.72)	
Brown-headed Cowbird (males only)	0.47	0.82 (1.13)	
Blue-winged Warbler (*Vermivora pinus*)^b^	0.58	0.78 (0.79)	Yes
Yellow Warbler (*Dendroica petechia*)	0.52	0.74 (0.88)	
American Goldfinch (*Carduelis tristis*)	0.41	0.62 (0.91)	
Mourning Dove (*Zenaida macroura*)	0.40	0.58 (0.84)	
Black-capped Chickadee (*Poecile atricapillus*)	0.39	0.51 (0.75)	
Tufted Titmouse (*Baeolophus bicolor*)	0.30	0.39 (0.66)	
American Robin (*Turdus migratorius*)	0.29	0.38 (0.64)	
Indigo Bunting (*Passerina cyanea*)^b^	0.30	0.35 (0.60)	Yes
Northern Cardinal (*Cardinalis cardinalis*)	0.30	0.33 (0.54)	
Chestnut-sided Warbler (*Dendroica pensylvanica*)^b^	0.28	0.32 (0.55)	Yes
Cedar Waxwing (*Bombycilla cedrorum*)	0.18	0.32 (0.77)	
Baltimore Oriole (*Icterus galbula*)^b^	0.25	0.30 (0.62)	
Red-eyed Vireo (*Vireo olivaceus*)	0.19	0.20 (0.43)	
Chipping Sparrow (*Spizella passerina*)	0.16	0.20 (0.50)	
Brown-headed Cowbird (females only)	0.15	0.20 (0.54)	
Black-and-white Warbler (*Mniotilta varia*)^b^	0.18	0.18 (0.39)	
Rose-breasted Grosbeak (*Pheucticus ludovicianus*)^b^	0.17	0.18 (0.42)	
Red-winged Blackbird (*Agelaius phoeniceus*)^b^	0.12	0.17 (0.56)	
Scarlet Tanager (*Piranga olivacea*)	0.14	0.16 (0.42)	
Tree Swallow (*Tachycineta bicolor*)	0.13	0.16 (0.47)	
White-eyed Vireo (*Vireo griseus*)	0.13	0.14 (0.38)	Yes
White-breasted Nuthatch (*Sitta carolinensis*)	0.12	0.13 (0.37)	
Red-bellied Woodpecker (*Melanerpes carolinus*)	0.11	0.11 (0.31)	

a Based on [35].

b Early successional species that are experiencing significant declines along Breeding Bird Survey routes in North America, 1966–2007 [14].

### Nest success

We located 55 prairie warbler nests during the summers of 2003, 2006 and 2007. Twenty-one field sparrow and nine eastern towhee nests were located in 2006–2007. For prairie warblers, the estimated probability of a nest surviving from laying through fledging was 17.4% (standard error - ±2.2%) in 2006 and 19.0% (±1.5%) in 2007; no nests were successful in 2003. The estimated probability of a field sparrow nest surviving from laying through fledging was 20.6% (±3.3%) in 2006 and 12.5% (±2.9%) in 2007. Although the probability of a nest surviving to fledging was low for these species, 46% of prairie warbler females and 71% of field sparrow females successfully fledged young (this included renesting) in 2006. In 2007, 28% of prairie warbler and 50% of field sparrow females produced fledglings. Eastern towhee nests had a 15.5% (±3.2) chance of surviving from laying through fledging.

For all species predation appeared to be the main reason for nest failure. At these nests, well-attended eggs or healthy nestlings that were too young to have fledged disappeared. Potential nest predators recorded weekly at the study site included eastern chipmunks *(Tamias striatus)*, blue jays *(Cyanocitta cristata)* and American crows *(Corvus brachyrhynchos).* In 2003, 2.6 chipmunks/km/day were detected during weekly transect surveys compared to an average of 0.3 in 2006 and 0.1 in 2007. In 2006, we saw 0.3 blue jays/km/day and no crows. In 2007, we saw 0.4 blue jays/km/day and 0.2 crows/km/day. Other potential predators observed at the site include common raven *(Corvus corax),* gray squirrel (*Sciurus carolinensis*), white-footed mice (*Peromyscus leucopus*), black racer (*Coluber constrictor)*, coyote *(Canis latrans)* and raccoon (*Procyon lotor).*

Brown-headed cowbirds, a brood parasite, were regularly observed at the site, often atop utility poles. Cowbirds laid eggs in the nests of prairie warblers (31% of nests) more frequently than those of field sparrows (10% of nests). Four prairie warbler nests failed directly as a result of brown-headed cowbird parasitism and the rate of parasitism on prairie warbler nests increased at the site from 14% in 2003 to 35% in 2006 and 41% in 2007.

In 2006 and 2007, the majority of prairie warbler nests that were parasitized by brown-headed cowbirds were located at the more heavily developed northern end of the nest study site. Parasitized nests were significantly closer to the nearest road and associated buildings than nests that were not parasitized (t = 4.79, df = 37, p<0.01).

Using values for prairie warbler nest success rates, the number of fledglings per successful nest, and juvenile and adult survivorship estimates, we calculated λ, the finite rate of increase for a population, to be 0.81 in 2006 and 0.76 in 2007. For field sparrows, λ was 0.81 in 2006 and 0.72 in 2007. These values indicate that the reproductive rates were insufficient to balance estimated losses from mortality in both of these years as well as in 2003 when none of 11 prairie warbler nests were successful.

### The effect of ROW and landscape characteristics on bird distributions

We ran three Poisson regression models (Table 2) for the abundance of each of six shrubland bird species and brown-headed cowbird females, and linear regression models for the number of shrubland bird species per plot. For field sparrow and the number of shrubland species, a best model was identified (Table 3, delta AIC of runner-up models were >2). For prairie warbler, indigo bunting, blue-winged warbler, eastern towhee, chestnut-sided warbler, and brown-headed cowbird females, delta AIC values and Akaike weights indicate substantial support for two or three models. For these species, variables with high positive regression coefficients (or low negative regression coefficients; independent variable data were standardized prior to modeling) in the best models were also present in runner up models. Table 4 lists the variables that had the most influence on abundance of particular species (regression coefficients of >0.25 or <−0.25 for models with delta AIC values of <2). For eastern towhee, no plot, ROW, or landscape scale variables had a strong effect on abundance. Deviance goodness of fit tests indicated that all models adequately fit the data. An autocovariate was included in the models for prairie warbler, indigo bunting, and chestnut-sided warbler; but spatial autocorrelation was still detected amongst the residuals for indigo bunting and chestnut-sided warbler.

**Table 2:** Parameters used in *a priori* models for analyzing the distribution of shrubland specialist birds and brown-headed cowbird.

	Shrubland species			Brown-headed cowbird (female)		
	model 1	model 2	model 3	model 1	model 2	model 3
Year	x	x	x	x	x	x
ROW width^a^	x	x	x	x	x	x
Vegetation height^b^	x	x	x	x	x	
Vegetation diversity		x				
Relative cover of grass/sedge		x				x
Relative cover of invasives^c^	x					
Relative cover of decid. erica. ssp.^d^	x					
Relative cover of *Kalmia latifolia*			x	x		
Total vegetation cover			x	x	x	x
Area of agriculture – 1 km^e^	x		x	x		x
Area of agriculture – 5 km^f^		x	x		x	
Area of development – 1 km^g^	x	x	x	x	x	x
Species specific autocovariate^h^	x	x	x			
Abundance of prairie warbler				x		x
Abundance of eastern towhee						x
no. birds per plot					x	

a quadratic for eastern towhee.

b natural log for no. shrubland species and chestnut-sided warbler.

c natural log for prairie warbler and indigo bunting.

d deciduous ericaceous species.

e quadratic for no. of shrubland species and blue-winged warbler, natural log for chestnut-sided warbler.

f natural log for eastern towhee and blue-winged warbler.

g natural log for eastern towhee, brown-headed cowbird, and the no. of shrub species.

h A species-specific autocovariate was used for prairie warbler, indigo bunting, and chestnut-sided warbler.

**Table 3:** AIC, delta AIC, and Akaike weight for *a priori* models. AIC values in bold identify models that received substantial support.

		model 1	model 2	model 3
Prairie Warbler	AIC	**230.33**	**229.79**	**230.69**
	delta AIC	0.54	0.00	0.90
	Akaike Weight	0.32	0.42	0.27
Indigo Bunting	AIC	**132.03**	134.07	**132.85**
	delta AIC	0.00	2.04	0.82
	Akaike Weight	0.49	0.18	0.33
Blue-winged Warbler	AIC	**211.72**	218.44	**211.75**
	delta AIC	0.00	6.72	0.03
	Akaike Weight	0.50	0.02	0.49
Eastern Towhee	AIC	**252.78**	**253.99**	**253.76**
	delta AIC	0.00	1.21	0.98
	Akaike Weight	0.46	0.25	0.28
Field Sparrow	AIC	227.39	224.63	**221.72**
	delta AIC	5.67	2.91	0.00
	Akaike Weight	0.05	0.18	0.77
Chestnut-sided Warbler	AIC	**130.71**	**130.14**	**131.66**
	delta AIC	0.57	0.00	1.52
	Akaike Weight	0.34	0.45	0.21
No. Shrubland Species	AIC	**332.99**	345.78	337.73
	delta AIC	0.00	12.79	4.75
	Akaike Weight	0.91	0.00	0.09
Brown-headed Cowbird	AIC	**100.21**	107.21	**98.24**
(females)	delta AIC	1.97	8.97	0.00
	Akaike Weight	0.27	0.01	0.72

**Table 4:** Independent variables with regression coefficients >0.25 or <−0.25 for models with delta AIC<2.

	Positive Relationship	Negative Relationship
Prairie Warbler	Autocovariate term	ROW width
Indigo Bunting	Year	Vegetation height
	ROW width	Development - 1 km
	log of invasive species	Relative cover of *Kalmia latifolia*
Blue-winged Warbler		Relative cover - deciduous ericaceous shrubs
		Quadratic of agriculture - 1 km
		Relative cover of *Kalmia latifolia*
Field Sparrow		ROW width
		Relative cover of *Kalmia latifolia*
		Development - 1 km
		Agriculture - 5 km
Chestnut-sided Warbler	Log of vegetation height	ROW width
	Autocovariate term	Development-1 km
	Total vegetation	
	Vegetation diversity	
Number of shrubland species	Relative cover of invasives ssp.	Quadratic of agriculture - 1 km
		Log of development - 1 km
Brown-headed Cowbird	Log of development - 1 km	ROW width
	Abundance of prairie warbler	Agriculture - 1 km
		Agriculture – 5 km
		Year

**Figure 2:**
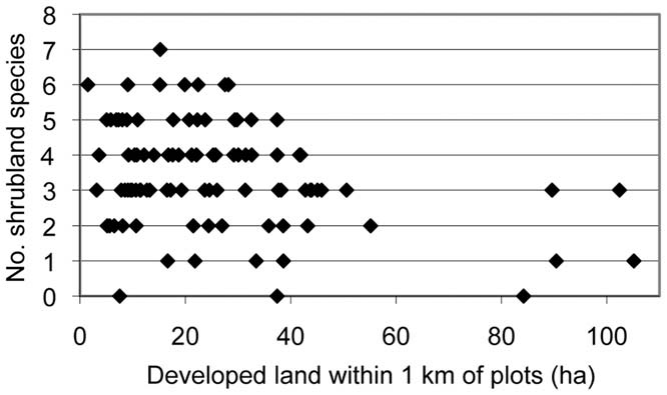
Relationship between the number of shrubland bird species per plot and the area of developed land within 1 km of a survey plot.

An increase in the area of developed or agricultural land in the surrounding landscape had a negative relationship with the number of species of shrubland birds (Figure 2) and the abundance of all the shrubland species except prairie warbler (Table 4) and eastern towhee. However prairie warbler had a strong positive relationship with the autocovariate term, which was negatively correlated with the area of agriculture within 1 km of a plot (Pearson's r = 0.60) and year (Pearson's r = 0.52). The species-specific autocovariate terms were not strongly correlated with any other independent variables.

Variables that describe conditions within the powerline corridor also influenced abundance (Table 4). For several species, a negative relationship with ROW width was detected, while vegetation height had a positive relationship with chestnut-sided warbler abundance and a negative relationship with indigo bunting abundance. In addition, the abundance of indigo bunting, blue-winged warbler, and field sparrow and the number of shrubland species were related to the relative coverage of certain types of vegetation. The autocovariate term was important for prairie warbler and chestnut-sided warbler (indicating the abundance of a species at a plot was correlated with abundance in adjacent plots). The abundance of brown-headed cowbird was positively related to prairie warbler abundance and development in the 1 km buffer and negatively related to year, ROW width, and agriculture in the 1 km and 5 km buffers.

## Discussion

### Powerline corridors as habitat for shrubland birds

Our results for point count surveys were generally consistent with the results of other studies of the distribution of birds on powerline corridors in the northeastern United States [7], [17], [15], [31]. We detected high densities of early successional birds that are of conservation concern in ROWs, including several species that have shown substantial continental declines on Breeding Bird Survey routes [14]. Also, one of these species, prairie warbler, had a higher density of territories in our nest-success study area (2.5 and 3.2 territories/ha in 2006 and 2007, respectively) than at any of the 32 study sites described by Nolan [32]; the highest density reported by Nolan was 2.0 territories (pairs)/ha on a powerline ROW in Maryland.

Although shrubland species were abundant in Connecticut ROWs, the estimated probabilities of nests surviving to fledging for prairie warbler, field sparrow and eastern towhee were less than 21%, which is low compared to rates observed for shrubland birds in other ROWs and in silvicultural openings. King and Byers [33] studied chestnut-sided warblers at two ROWs in western Massachusetts and reported an 83% probability of nest success. Confer and Pascoe [7] found the probability of shrubland bird nests surviving to fledging was 55% on ROWs in heavily forested regions in New York, Massachusetts and Maine. Nest success ranged from 43% to 99% in studies of shrubland birds in clearcuts in heavily forested regions of New England [34]–[35], and from 35 to 65% for four species of shrubland birds in fields managed for conservation in Connecticut [36]. However, the probability of nest success at our study site was similar to those calculated by Kubel and Yahner [37] for golden-winged warblers (*Vermivora chrysoptera*) (20%) in a powerline corridor in Central Pennsylvania and by King et al. [23] for shrubland birds in several ROWs in western Massachusetts (13.9%). (The latter mean included data from ROWs that were narrower than the ones in our study, however, and these had much lower average nest success rates compared to wider corridors in the same region.) Also, Nolan's intensive study of prairie warblers breeding in old fields in Indiana between 1952 and 1962 revealed nest success rates of only 20–22% [32].

Although predation was the primary reason for low nest success at our study site, brood parasitism also played a role. The percent of prairie warbler nests parasitized by cowbirds in 2006 and 2007 was higher than reported in other studies of shrubland birds in powerline corridors. King and Byers [33] found that only 2% of the nests of chestnut-sided warblers were parasitized by cowbirds, while Confer and Pascoe [7] and Meehan and Haas [38] observed parasitism rates on nests of various species of 5.3% and 4.7%, respectively. All of these sites were in heavily forested regions, while only 58% of the landcover within 10 km of our site was forested.

As a result of low nest success, productivity at our site was apparently insufficient to compensate for estimated annual mortality for both prairie warblers and field sparrows. However, as in most previous studies, the estimates of survival rates used in calculating λ are approximate. Although we were able to use especially robust data on both juvenile and adult survivorship for prairie warbler from Nolan's [32] long-term study, these data were from another region and time period, as were the estimated survival rates for field sparrows. Determining survival rates for adults and juveniles in Connecticut powerlines would improve the accuracy of our λ calculations.

Although our site had relatively low nest success rates in the three years we monitored nests, this might not always be the case [39]. In some populations, low reproductive success in some years is compensated for by high reproductive success in other years [40]. A range in the percent of successful nests in different years was documented for field sparrows (20–63%; [41]) and prairie warblers (12–35%; [32]).

If the number of young fledging each year is insufficient to compensate for annual adult mortality in these species, then the local population may be sustained by immigration of individuals from other sites. The study site would act as a population sink but would still contribute to the larger regional population by supporting adult birds during the breeding season and by producing some offspring. Also, the local population can remain stable as long as other populations produce a surplus of young to provide immigrants.

### Relationships between habitat characteristics and the distribution of shrubland birds

The abundance of particular species of shrubland birds was related to habitat characteristics of the ROW study plot as well as characteristics of the regional landscape surrounding the plot. Several species showed positive or negative relationships with the percent cover of particular types of plants, indicating that different species of birds are favored by different kinds of shrubland vegetation [23], [42]–[43]. Also, the abundance of indigo bunting showed a negative relationship with vegetation height. Shrubland birds generally decline as succession occurs and vegetation becomes taller, a pattern that has been demonstrated in studies of clearcuts [35]. In contrast, chestnut-sided warblers tend to be more abundant at sites with taller vegetation. The range of vegetation heights is restricted along powerline corridors because of regular maintenance to limit the height of vegetation and prevent power outages. Consequently, it is not surprising that vegetation height is not an important predictor for some shrubland species nesting along powerlines. However, the height and species composition of vegetation on powerline ROWs could be managed to favor particular species that have a high priority for conservation.

Another plot-level characteristic, the width of the powerline corridor, was negatively related to the relative abundance of prairie warblers, chestnut-sided warblers and field sparrows, and positively related to the abundance of indigo buntings. As with previous analyses of the effect of ROW width on abundance of birds [7], [23], we compensated for the area of shrubland habitat available in corridors of different widths when analyzing bird distributions. Confer and Pascoe [7] also found a negative relationship between ROW width and the abundance of several species of shrubland birds, including prairie warbler and field sparrow. Anderson et al. [44] found a negative relationship between ROW width and abundance for prairie warbler, and a positive relationship for field sparrow. In a recent study of ROW birds in central Massachusetts, King et al. [23] found positive relationships between ROW width and abundance for several species of shrubland birds, but these relationships were actually quadratic. Abundance increased with width up to an intermediate width and then stabilized or declined on wider corridors (see Figure 2 in [23]). King et al. studied relatively narrow corridors (15–78 m wide) compared to our sites (43–155 m), so we may have been analyzing data from the opposite side of a parabolic, quadratic curve. A complex quadratic relationship between abundance and corridor width may explain why some studies yield positive relationships for ROW width while others yield negative relationships for the same species on different sets of ROWs.

Landscape-level variables were also important predictors of the abundance of shrubland birds. The abundance of several species was related to the amount of agriculture or development in the surrounding region; these species were less common in landscapes with a lower proportion of forest. The abundance of indigo buntings and field sparrows declined as the amount of residential/commercial development near survey plots increased, and all other shrubland species were less abundant as the amount of agriculture increased.

Only brown-headed cowbird showed a positive relationship with development, and higher rates of brood parasitism by cowbirds in developed areas may be one reason that shrubland birds are less frequent on powerlines in these areas. Brown-headed cowbird abundance was greater at sites with a greater abundance of prairie warblers, which, based on our nest study, may be an important host species for this brood parasite. Like prairie warblers, cowbirds show a negative relationship between abundance and corridor width. A negative relationship between amount of agriculture within 1 km and 5 km and number of brown-headed cowbirds is surprising given their strong association with farmland and feedlots [45], but in coastal Connecticut suburban residential areas may be a more important habitat for cowbirds than are farming areas.

### Forest fragmentation and nest success in shrubland birds

Although shrubland birds may not be sensitive to fragmentation of their preferred breeding habitat [35], [46]–[47], ironically they may be affected by the amount of fragmentation of the mature forest in which a shrubby opening is embedded. In the northeastern United States, shrubland habitats are often restricted to small patches surrounded by mature woodland. The amount of forest cover in the surrounding landscape probably determines the density of predators and brood parasites (cowbirds) within these small patches. Mature-forest birds nesting in landscapes with extensive development and forest fragmentation generally suffer higher rates of nest predation and brood parasitism than do those nesting in landscapes with unbroken forest [48]. Our results show that shrubland birds nesting in an area with moderate residential development had relatively low rates of nest success compared to rates documented in previous studies in more heavily forested regions. Moreover, the potential importance of regional forest cover is indicated by the lower abundance of some species of shrubland birds on ROW plots in landscapes with a higher proportion of residential/commercial development or farmland and a lower proportion of forest cover. The implication is that the most valuable ROW habitat for shrubland birds may be in regions in which the powerline corridor is surrounded by extensive, continuous forest. Schlossberg et al. [27] found that the abundance and nest success of most shrubland bird species were unaffected or positively affected by the amount of development within 1 km of study sites, while only two shrubland species showed negative relationships with amount of development and abundance or nest success. Their study was completed in a heavily forested region in which only 4% of the entire region is developed, however. Our results are consistent with those of Burhans and Thompson [26] who found lower abundance of some shrubland bird species and higher rates of parasitism by cowbirds (but not higher rates of nest predation) in urban landscapes than in rural landscapes in a region with 17% overall development. The amount of development in the four watersheds where our survey plots were located ranged from 7 to 20% [49], but was as high as 33% within a kilometer of some survey plots.

### Conclusions

This study and previous studies in eastern North America have shown that shrubland birds achieve high densities on powerline ROWs that are managed by selective removal of trees to establish relatively stable vegetation dominated by low shrubs. This approach could be applied to other early successional forest habitats in order to sustain regional biological diversity, and it could be tested on utility corridors in other parts of the world, particularly in regions of East Asia and Europe [28]–[30] where some scrub/shrub species are declining. In contrast to studies of ROWs in more heavily forested regions, however, we found relatively low nest success rates for our two focal species. In light of these results, bird populations in powerline corridors in more heavily settled areas should be studied carefully to determine whether or not they are sink populations. The best sites for shrubland bird conservation may be on corridors in less heavily developed regions, particularly corridors that traverse large, protected forests. Creation of new powerline corridors through heavily forested regions is not generally recommended, however, because it results in forest fragmentation that may have a negative effect on birds nesting in the surrounding forest [50].

## Materials and Methods

### Ethics statement

Permission to complete surveys of plants and birds on powerline corridors was obtained from Anthony Johnson III, Supervisor of Transmission Vegetation Management, Northeast Utilities. We obtained a permit to monitor bird nests from the Connecticut Department of Environmental Protection (Permit Number 0109003b, issued January 31, 2007). The Connecticut College Institutional Animal Care and Use Committee did not require an Animal Use Permit for this study because it was an observational field study that did not involve capturing animals or maintaining animals in captivity.

### Shrubland bird surveys

During the breeding seasons of 2003, 2006, and 2007 we conducted bird surveys and vegetation transects on 93 plots along powerline ROWs owned by Northeast Utilities in southeastern Connecticut. We surveyed all ROWs within this region that were wide enough to have shrubland habitat and where we could obtain permission. The width of ROW corridors ranged from 43 to 155 m (average = 84 m±2.4 [SE]). Different plots were examined each year. Plots were located every 200 m in seven separate sections of rights-of-way that stretched for 1–3 km without interruption by roadways or residential areas. All but one of these sections were part of an interconnecting web of powerline corridors. Each section contained 5–20 survey plots, and all plots were within 50 km of one another.

Each plot was visited twice, once between June 1 and 15 and again between June 16 and July 5, to complete bird surveys. At least two weeks passed between visits. Surveys were conducted between 06:00 and 10:00 Eastern Daylight Time, and were not performed when winds exceeded 16 km/h or precipitation was more than a light drizzle. During each visit two observers recorded all birds seen or heard within a 50-m radius of a survey point during a 10-min period [51]–[52]. To ensure that we did not count the same individual bird twice during a survey, we only counted individuals of the same species as separate individuals if we detected them simultaneously or if they alternated songs or calls repeatedly from widely separated locations. In the analyses, the abundance for each species was defined as the maximum number of individuals detected during either of the two survey periods. This procedure yields an index of abundance rather than an accurate estimate of density, but this is sufficient for analyzing the distribution of birds in a large-scale survey in one general type of habitat [53], and does not introduce unnecessary biases from distance sampling for birds that are primarily detected by sound [54]–[55].

Vegetation sampling was performed between late June and early July on a line intercept transect [56] originating at the center of the plot and stretching out 25 m in a compass direction generated with a random number table. The length of survey tape intercepted by each plant taxon, including foliage that overlaid the line, was recorded. Vegetation height was measured at 0, 5, 10, 15, and 20 m along the transect.

Land cover surrounding each plot was measured using Arc GIS 9.2 (Environmental Systems Research Institute, Redwood, CA). Land cover maps for southeastern Connecticut were downloaded from the University of Connecticut's Center for Land Use Education and Research [49]. Buffers of 1 and 5 km were generated for each plot so that we could calculate the area of each buffer covered by forests, developed areas (commercial, industrial, and residential areas, as well as adjacent roads and maintained grassy areas), and agricultural areas (non-maintained grassy areas, pastures, and croplands).

### Analysis of bird distributions

Three *a priori* models were developed for each shrubland specialist with an adequate sample size (Table 1); the number of shrubland species; and female brown-headed cowbirds, which are brood parasites. We employed log-linked Poisson regression to calculate Akaike's Information Criterion (AIC) for models using the statistical package R [57] for shrubland species and cowbirds. Linear regression was used for the number of shrubland species, which was normally distributed. Delta AIC and Akaike weights were calculated to facilitate model comparison and a deviance goodness-of-fit test was used to assess the fit of the models to the data for each species. (An R^2^ statistic was used for number of shrubland species.) We used number of shrubland bird species per plot and the abundance of prairie warblers, eastern towhees, field sparrows, blue-winged warblers, indigo buntings, and chestnut-sided warblers and female brown-headed cowbirds as dependent variables. We chose independent variables that had previously known relationships with abundance of particular species or shrubland birds in general (Table 2). Vegetation diversity was calculated using the Shannon diversity index with the relative coverage data generated from each transect [58]. Also, the year a plot was surveyed was included in all analyses to account for variation in abundance among years. A species-specific autocovariate was added to a model when a Moran's I test detected spatial autocorrelation amongst the model residuals [59]. We computed autocovariates based on the distance between plots and the abundance of a given species [60]. Pearson's r was employed to identify correlations between the autocovariate terms and other independent variables.

We used ln or quadratic transformations of independent variables in the regression model when the transformed variable appeared to have a clearer relationship with the dependent variable in a scatterplot. We also calculated Pearson's r to determine how strongly independent variables were correlated with each other. Because of strong correlations among the areas of forested and developed land in the 1 and 5 km buffers, the only land-use variables we included in the analyses were the area of developed land at 1 km and the area of agricultural land at 1 and 5 km.

We normalized the independent variables by converting to z-scores so that regression coefficients could be compared. In addition, we included the natural logarithm of the amount of shrubland habitat in each plot as an offset in the regressions in order to standardize for the amount of appropriate habitat for shrubland specialists that was sampled. On wide corridors, the entire plot was within the powerline corridor (and hence was characterized by open, shrubby vegetation), but on narrow corridors the plots extended into the adjacent forest so a smaller area of open habitat was sampled. By standardizing the area of corridor habitat included in the surveys, we ensured that any relationships with corridor width were not an artifact of sampling a smaller area of appropriate habitat on narrow corridors.

### Monitoring nest success

During the summers of 2003, 2006 and 2007, we mapped prairie warbler and field sparrow territories and monitored nests along 1 km of Northeast Utilities powerline 383/310 in Montville, Connecticut. We visited the site 3–5 times per week from mid May until late July. During each visit, the locations of all prairie warblers (2003, 2006 and 2007) and field sparrows (2007) detected by sight or sound were mapped. Sex, location, movements, vocalizations, and interactions with other birds were documented for each individual to help determine its territorial and mating status. Territorial boundaries were estimated from the positions of counter-singing males and territorial encounters [61]. Once each week we conducted a transect survey of potential nest predators (corvids and eastern chipmunks) in the study area; the starting point alternated from week to week between the northern and southern ends of the site.

We primarily searched for and monitored prairie warbler and field sparrow nests, but we also monitored nests of any other early successional species. Nests were checked every 3–4 days and were always approached from a different direction to avoid leaving a trail that predators might follow. The number of conspecific eggs or nestlings plus those of brown-headed cowbirds was recorded along with the age of the nestlings. A nest was deemed successful if fledging was observed or if fledglings were seen in the territory after the estimated date of fledging. A nest was considered a failure if only cowbirds fledged or if eggs/nestlings disappeared prior to the estimated date of fledging.

### Analysis of nest success

To facilitate comparison with previous studies of nest success of shrubland birds, the Mayfield method [62] was employed to calculate the probability of nest success. We counted the number of days nests were observed during the laying, incubation, and nestling phases, including days when the eggs were being laid only if nest building was witnessed. We used the Last Active-B approach [63] to estimate the end of the observation period; this method performs well whether or not daily mortality rates for eggs and nestlings are constant. The length of the laying phase and the probability that an egg would hatch were calculated from the data. We used the length of the incubation and nestling phases reported in Nolan [32] for prairie warblers and Carey et al. [41] for field sparrows. The variance and standard error for the probability of nest success were calculated using the equations in Johnson [64].

To examine whether this site acts as sink or source habitat, we calculated λ, the finite rate of increase for a population, as in Flaspohler et al. [65]. Lambda is the annual adult survival rate plus the product of per capita annual production of female fledglings and annual juvenile survival rate. A value of λ>1 indicates that the site is source habitat where reproduction is more than sufficient to balance mortality, while a value of λ<1 corresponds to sink habitat [33]. We used the fledgling and adult survival statistics from Nolan [32] for prairie warblers and from Carey et al. [41] for field sparrows. We used an equation for per capita annual productivity (F) that assumes birds consistently renest after unsuccessful nest attempts and that there is a 1∶1 sex ratio for nestlings [65]. For field sparrow, a slightly different formula was used that incorporated a 50% chance (derived from our data) of renesting following a successful first nest. In calculating F we used the average number of fledglings per successful nest in place of average clutch size to account for hatchling mortality.
